# Prevalence of T-2 Toxin in the Food and Beverages of Residents Living in a Kashin–Beck-Disease Area of Qamdo, Tibet

**DOI:** 10.3390/nu16101449

**Published:** 2024-05-11

**Authors:** Tong Jiang, Junan Yan, Hongxing Tan, Zhu Pu, Ou Wang, Tao Liu, Zhaoyu Chen, Jiaxiang Gao, Jun Wang, Jianhao Lin, Junsheng Huo, Jian Huang

**Affiliations:** 1 National Institute for Nutrition and Health, Chinese Center for Disease Control and Prevention, Beijing 100050, China; jiangtong@ninh.chinacdc.cn (T.J.);; 2 Key Laboratory of Public Nutrition and Health, National Health Commission of the People’s Republic of China, Beijing 100050, China; 3 Shenzhen Chronic Disease Prevention and Treatment Center, Shenzhen 518000, China; 4 Center for Disease Control and Prevention, Luolong County, Chamdo 855400, China; 5 The Department of Orthopedics & Traumatology, Peking University People’s Hospital, Beijing 100044, China

**Keywords:** Kashin–Beck disease, T-2 toxin, Tibet, brick tea, selenium

## Abstract

It has been strongly suggested that selenium deficiency and T-2 toxin contamination have a strong relationship with the occurrence and development of Kashin–Beck disease (KBD). In order to provide information for understanding the high prevalence of KBD in Tibet, this study collected the responses to a cubital venous blood and dietary questionnaire of 125 subjects including 75 KBD patients and 50 healthy controls in a KBD-prevalent county (Luolong County) in Tibet, China. A total of 10 household local families were randomly selected in this area, and local diet samples of brick tea, Zanba powder, milk residue, and hulless Barley were collected from these residents. Selenium content in blood was detected by inductively coupled plasma mass spectrometry (ICP-MS). The T-2 toxin contamination level in food sample was assayed using an ELISA kit. The selenium levels of patients and controls were 42.0 ± 19.8 and 56.06 ± 22.4 μg/L, respectively. The serum selenium level in controls was higher than that in patients, but there was no significant difference, and the serum selenium level both in patients and controls in Tibet was lower than the normal range. The results of the dietary survey showed that the number of respondents who consumed butter tea was large; 46.67% of patients indicated that they drank buttered tea every day, which was significantly higher than in controls. The contents of T-2 toxin in Zanba powder, milk residue, hulless barley and drinking water samples were below the detection limit (0.05 μg/kg); this result was labeled Tr. Unexpectedly, the contents of T-2 toxin in brick tea were higher, with average levels of 424 ± 56 μg/kg in Detong village and 396 ± 24 μg/kg in Langcuo village. For the first time, we report the presence of an extremely high concentration of T-2 toxin in brick tea of Tibet.

## 1. Introduction

Kashin–Beck disease (KBD) is an endemic osteoarthropathy distributed throughout North Korea, Siberia, Japan, and China. The main pathogenic sites of KBD are irreversible coagulation necrosis and apoptosis in chondrocytes from articular cartilage, epiphyseal cartilage, and epiphyseal plate cartilage. It is more common in the adolescent population at the stage of chondrodevelopment [1]. The name Kashin–Beck commemorates two physicians, Nicolai Ivanowich Kashin (1825–1872) and Eugene V Beck (1885–1916), for their contribution to this particular type of osteoarthropathy [2,3]. In the 21st century, this condition has been most prevalent in China. Endemic areas encompass 13 provinces (or city/autonomous regions) and 379 counties from the northeast to the southwest. Although KBD is essentially controlled in China at present, new clinical cases still emerge, especially in endemic zones in Tibet [4]. According to data up to 2020, approximately 9122 people (clinical degree class I and above) in 178 townships of 54 counties (73% of counties) in Tibet are still facing adversity linked to KBD. The most affected areas are Bianba County and Luolong County of Qamdo City. The cases in these two counties are distributed across all age groups, and the clinical detection rate of Kashin–Beck disease in some villages has reached more than 70 percent, indicating that Kashin–Beck disease has a long history as an epidemic in the local area [5]. According to the latest data released in 2019, the prevalence of Kashin–Beck disease in Tibet from 1999 to 2016 was characterized by the following factors. (1) A wide age distribution: the youngest patient was less than 4 years old and the oldest patient was 75 years old, and the disease was grade III, indicating that Kashin–Beck disease has been prevalent on the Tibet Plateau for more than 100 years. (2) A long duration: patients in all age groups can be seen with degrees Ⅱ and Ⅲ, indicating a long time during which the epidemic has been active and serious. (3) A wide spatial distribution, from the former to the latter, and spread to the surrounding areas (Qinghai, Gansu, parts of Sichuan, etc.). (4) There is an altitude correlation. Almost all the Kashin–Beck disease areas on the Tibet Plateau exist above 2000 m, and the cases of Kashin–Beck disease are very mild in areas below 2000 m. (5) The disease is related to grain, all affected areas are concentrated in agricultural areas, and there is no Kashin–Beck disease in pastoral areas. (6) The incidence of family cluster is present. The Qamdo region of Tibet is an area that is seriously affected by Kashin–Beck disease. For many years, Kashin–Beck disease has seriously affected the health of people in the affected area and seriously restricted the local economic development. Therefore, Kashin–Beck disease in Qamdo region of Tibet is still a difficulty and a key point that needs continuous attention, research, and a solution [6].

At present, the most established theory suggests selenium deficiency and T-2 toxin as the causes of KBD. Several factors, including nutritional deficiency and ingesting of grains with mycotoxins, have been reported to be associated with KBD [7]. Kashin–Beck disease is defined as “poor disease” in a certain sense, and its occurrence and development degree are related to local economic conditions and family living standards. Many studies have pointed out that with an improvement in living conditions and diversification of diet in the affected areas, the prevalence of Kashin–Beck disease has been controlled to varying degrees in different affected areas, and some affected areas have been transformed into non-affected areas, which also indicates that Kashin–Beck disease has a certain correlation with diet and nutrition [8]. T-2 toxin is the most toxic of the trichothecenes, which is a group of mycotoxins produced mainly by members of the Fusarium genus [9,10]. Studies have shown that T-2 toxin predisposes individuals to osteoarthritis and Kashin–Beck disease (KBD). The major pathological change associated with KBD is the degradation of the articular cartilage matrix. Mycotoxins are widely distributed in various food crops (wheat, rice, and corn) and semi-finished products (bread, etc.). Previous studies on fungi in Tibet found that barley grains in KBD-affected areas were more seriously contaminated by fungi than those in non-affected areas. T-2 toxin is a common form of mycotoxin contamination in food; it also has strong heat resistance and is not destroyed by conventional cooking heating, so it is considered to be a possible pathogenic factor of KBD [11,12,13]. In this study, the serum selenium levels and dietary intakes of patients and controls in the ward of Luolong County, Qamdo, Tibet, which is affected by Kashin–Beck disease, were reviewed and compared using the case–control method, and food and water samples of 10 households in the ward were collected randomly to detect the content of T-2 toxin. The results can help to provide new thinking for local disease control departments to prevent and intervene in KBD, and, hopefully, provide some new enlightenment regarding the pathogenesis of KBD.

## 2. Materials and Methods

### 2.1. Selection of Investigation Regions, Sites and Population

Tibet is located in northwest China. The national monitoring data of Tibet for KBD and the Criteria for Delimitation and Classification of Kashin–Beck Disease Endemic Areas (GB 16395-2011) [14] were used to select historical KBD endemic areas in Tibet for this investigation.

Patients who were screened and registered in the national Kashin–Beck disease surveillance system in 2021 in Luolong County, Tibet, were selected as the source population of the case group, and those who did not have Kashin–Beck disease after screening were randomly selected from the same county as the control group. Inclusion criteria: All subjects met the diagnostic criteria for Kashin–Beck Disease (WS/T207-2010) [15], and children aged 5 to 14 years had received right wrist orthography. Exclusion criteria: Residents who did not participate in the 2021 national KBD screening in Tibet, residents who could not confirm whether they had KBD or not, and residents with incomplete data entry.

### 2.2. Survey Objects and Blood Sample Collection

Blood sample collection: A total of 125 people (75 were patients and 50 were controls) who lived in the same area from a family of more than two generations (at least 3 people) who volunteered to be investigated in Luolong County, Qamdo, Tibet, were selected. In addition, 100 healthy people in Shenzhen, a non-KBD-disease area, were selected as blank controls for this study. All methods were carried out in accordance with relevant guidelines and regulations from China CDC NINH.

A self-made questionnaire was used to conduct a dietary survey, and the respondents were interviewed individually in October to November 2021, with the questionnaire completed on the spot. The alcohol and beverages consumed by the respondent’s family in the past year, the frequency of intake of each type of alcohol and beverage, and basic information about the respondent, family size, and per capita income were obtained. Investigators were trained professionally before the investigations. Subjects were strictly included according to the inclusion and exclusion criteria of the national diagnostic standard of KBD.

This study was approved by the Institutional Review Board (or Ethics Committee) of National Institute for Nutrition and Health, Chinese Center for Disease Control and Prevention (Approval NO. 2021-009), and all included participants provided informed consent before any activity was conducted.

### 2.3. Collection of Food Sample

In October 2021, a total of 42 food samples (8 samples of brick tea, 8 samples of Zanba powder, 8 samples of milk residue, 8 samples of hulless barley, and 10 samples of drinking water) were gathered from two KBD historically endemic villages (Detong and Langcuo villages) in Luolong County, Qamdo, Tibet. All collection food samples (not less than 10 g/household) were placed in envelopes and stored at −20 °C until analysis in order to avoid any contamination in the storage bags. Drinking water samples (not less than 20 mL/water source) from headwaters in the two KBD-endemic villages were collected using 50 mL sterile tubes and kept at 4 °C until use.

### 2.4. Determination of Selenium Content in Blood

For the analysis of whole blood Se, 1 g of a previously heated (25 °C) and shaken blood sample was weighed into 10 mL Teflon microwave vessels and 2 mL of 65% HNO_3_ was added. During digestion, the samples were digested at 120 °C for 10 min, after which the temperature was ramped to 120 °C (within 8 min), and then to 160 °C for 10 min, and finally to 175 °C for 20 min using a CEM MARS Xpress microwave system (CEM, Matthew, NC, USA). The cooled, digested samples were diluted to 10 mL with ultrapure water and analyzed for total Se content by inductively coupled plasma mass spectrometry (ICP-MS).

### 2.5. Determination of T-2 Toxin Contamination Levels in Food and Water Samples

A total of 1 g of finely ground food samples was extracted with 20 mL 60% methanol/H_2_O (*v*/*v*). The mixture was thoroughly blended using an oscillator at high speed for 5 min, and then centrifuged at 4000 rpm for 5 min. The supernatant was used for T-2 detection. The T-2 toxin content in the food samples was measured using an ELISA kit (Renjie Biotechnology Co., Ltd., Shanghai, China). The main detection steps were to take filtered or appropriately diluted samples, add 50 μL standard or sample per well, and then add 50 μL of HRP-conjugate to each well and 50 μL of antitoxin antibody to each ELISA reaction well. This was mixed and shaken well, then incubated at 25 °C for 30 min. Each well was aspirated and washed, with this process repeated 5 times. A total of 100 μL of reaction substrate was added for a 15 min reaction, and the ELISA reaction was terminated with the stop solution after incubation at 25 °C for 15 min. The optical density of each well was determined using a microplate reader set to 450 nm. The sample average absorbance values were compared with standard values, and the T-2 toxin concentration in the samples was determined. All the samples were measured in triplicate (independent aliquots), and the mean of the three aliquots was calculated to derive the T-2 toxin content of the food.

### 2.6. Statistical Analysis

All data were recorded using Excel 2016 software and all statistical analyses were performed using SPSS (version 22.0). The significance levels of the selenium and T-2 toxin contents were determined using a *t*-test and ANOVA. A two-sided *p* < 0.05 was considered statistically significant.

## 3. Results

### 3.1. Characteristics of the Population

The subjects of this survey were all Tibetans, including 75 males (38 patients and 37 controls) and 50 females (37 patients and 13 controls). The average age of patients was 51 ± 12 years old, and the average age of controls was 41 ± 11 years old.

### 3.2. Selenium Concentration in Blood of KBD Area and Non-KBD Area

**Figure 1:**
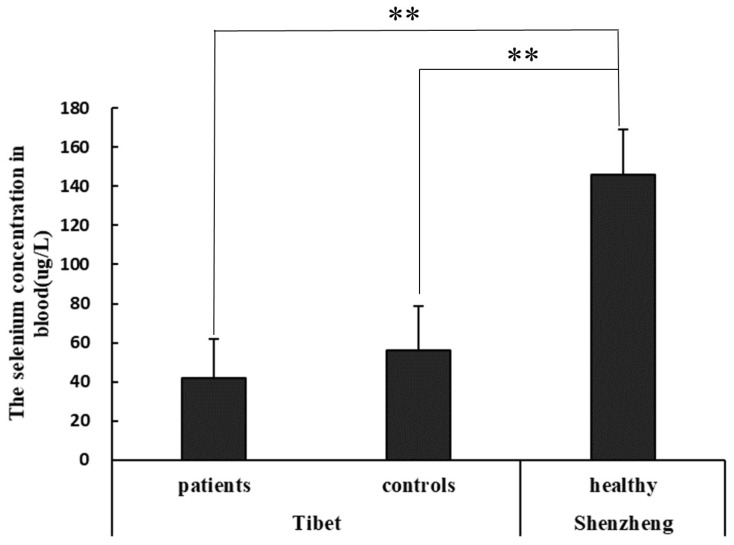
Blood selenium levels of healthy people in Shenzhen, and patients and controls of Kashin–Beck disease areas in Tibet (** represent a difference of significance of 0.1%).

The results indicated that the selenium concentration in Tibet (KBD-endemic areas) was at a low level. The average selenium content in KBD-endemic areas of patients and controls was 42.0 ± 19.8 μg/L and 56.06 ± 22.4 μg/L, respectively. The selenium level of patients was lower than that of controls, but there was no significant difference between patients and controls. The selenium content of patients and controls in Tibet was significantly lower than that of healthy people in Shenzhen (146.1 ± 23.0 μg/L), which is in a non-KBD area (Figure 1). According to the standard value range of human serum selenium of 100–200 μg/L, the serum selenium content of patients and controls in Luolong County, Qamdo, Tibet, was seriously below the standard value, indicating that there is still a common nutritional status of selenium deficiency among residents in historical KBD areas of Luolong County, Qamdo, Tibet.

### 3.3. Comparison of Drink and Beverage Dietary Intake and Frequency in KBD Area between Patients and Controls

**Figure 2:**
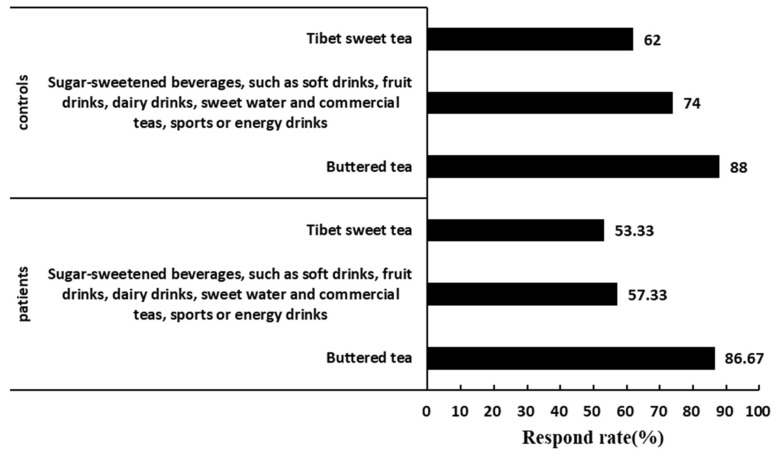
Response rate (%) for the top-three ranked drinks and beverages of patients and controls.

The dietary survey results showed that the top-three drinks and beverages among the dietary intake responses of both patients and controls of the KBD area of Tibet were buttered tea; sugar-sweetened beverages, such as soft drinks, fruit drinks, dairy drinks, sweet water and commercial teas, sports or energy drinks; and Tibet sweet tea (Figure 2). The consumption rate of butter tea in patients and controls were the largest, i.e., 86.6 7% and 88%, respectively. The consumption rate of coffee was significantly higher in the controls than in patients (Table 1).

**Figure 3:**
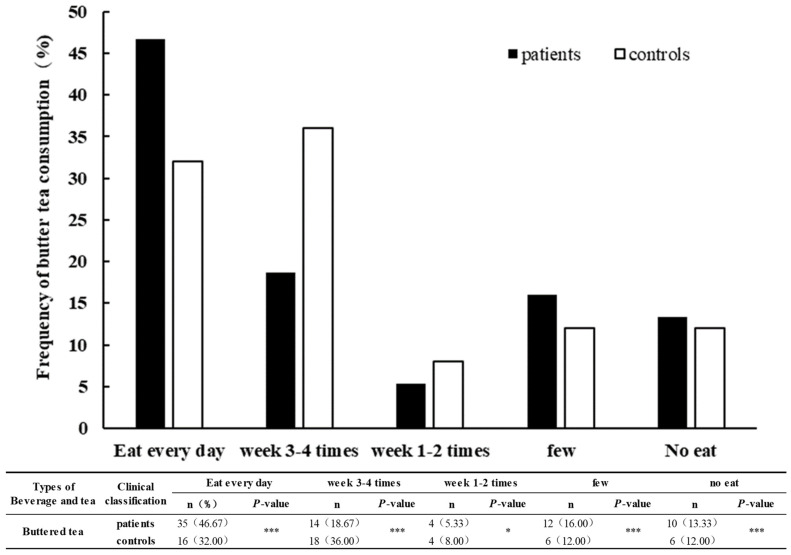
Frequency of butter tea consumption of patients and controls. * represents *p* < 0.05, a significant difference in people who responded between patients and controls. *** represents *p* < 0.001, a significant difference in people who responded between patients and controls.

The consumption frequency of butter tea intake in patients and controls showed that daily consumption of butter tea was reported in the largest number of patients, whereas among controls, the largest number of respondents indicated they consumed butter tea 3–4 times a week. Daily butter tea consumption of patients (46.67%) was reported significantly more than by controls (32%), and the consumption of butter tea 3–4 times a week reported by controls (36%) was significantly more than that of patients (18.67%) (Figure 3). As a whole, butter tea is an important drink for Tibetan people, and the frequency of butter tea intake, and its consumption, are high in Tibet. In brief, the consumption frequency of butter tea in patients was significantly higher than that of controls (Figure 3).

### 3.4. T-2 Toxin Contamination Level in Food and Drinking Water Samples

The contents of T-2 toxin in Zanba powder, milk residue, hulless barley, and drinking water samples (Table 2) were below the detection limit (0.4 μg/kg), and the T-2 toxin results were all undetected. The contents of T-2 toxin in brick tea were higher, with average levels of 424 ± 56 μg/kg in Detong village and 396 ± 24 μg/kg in Langcuo village, and T-2 toxin was detected in all of the collected tea samples. Food sample detection showed that the level of T-2 toxin was unexpectedly high in brick tea, and that T-2 toxin does not exist in grain in Tibet but does exist in tea.

## 4. Discussion

Tibet has been a historically serious area for Kashin–Beck disease. However, many studies have shown that no excess levels of mycotoxins (such as T-2 toxins) have been detected in grain in Tibet, and also no suspicious water quality risks exist [16]. Researchers published the results of the mycotoxin pollution distribution of five main grain and oil crops (hulless barley, wheat, rape, corn, and pea) in 16 counties of 6 prefectures and cities in Tibet. The limit of T-2 toxin was based on the standard of German barley, whose maximum content was limited to 6.0 μg/kg. T-2 toxin was not detected in five major grain and oil crops in 16 counties of 6 prefectures and cities in Tibet [17]. As reported by other researchers, no T-2 toxin was detected in hulless barley [16], Zanba powder, milk residue, or drinking water samples collected in Tibet [18]. Surprisingly, our investigation detected a very high content of T-2 toxin in brick tea samples from the Qamdo region of Tibet. Its content (396 ± 24~424 ± 56 μg/kg) was 66–70 times higher than the standard regarding German barley (6 μg/kg). We would like to point out here that because there is currently no specific published method for the detection of T-2 toxin in tea, we detected T-2 toxin in tea using the detection method used of cereals (such as peanuts, corn, oats, and soybeans) and other feed. This ELISA kit employs the competitive inhibition enzyme immunoassay technique. The results were verified by the recovery experiment of standard samples, from which the average recoveries were 80.8~95.7%, indicating that the method could be applied to the detection of T-2 toxin in tea. We hope that more methodological researchers can participate in the establishment of T-2 toxin detection methods in tea, and can establish a simple method specifically for the detection of T-2 toxin in tea samples, so as to obtain results more quickly and accurately.

Brick tea is a base material for making butter tea in Tibet, which a large number of Tibetan people drink every day. The harsh environment of the plateau has led to limited food sources for the local Tibetan people, who form the habit of drinking tea leaves as a satiation solution to digest greasy food and replenish vitamins [19]. The results of our dietary survey showed that the most consumed beverage, in both patients and controls in Qamdo, Tibet, was butter tea. The number of respondents who indicated they drank butter tea daily was largest in patients, and accounted for 46.7% of the total number of respondents. The number of controls who reported drinking three to four times per week was the highest, accounting for 36% of the respondents.

Researchers observed the effects of low selenium and T-2 toxin on cartilage joints in rats through modeling. It was found that low selenium and T-2 toxin accelerated the death of chondrocytes, and the results supported the relationship between T-2 toxin and Kashin–Beck etiology under low-selenium conditions [20]. Although it has been reported that Kashin–Beck disease may occur under the influence of the dual factors of low selenium and the presence of T-2 toxins [21], we still believe that a higher intake of T-2 toxins at low selenium levels may increase the risk of Kashin–Beck disease, although this is not the only necessary reason. Currently, we are carrying out whole genome detection of SNPs in patients and controls in areas in which Kashin–Beck disease is serious, hoping to find the differences in gene loci between patients and controls, so as to provide more scientific evidence to explain the occurrence of Kashin–Beck disease.

So far, there have been no clear reports on the per capita annual consumption of brick tea in Tibet. Local disease control and government departments indicate that the per capita annual consumption of tea in the Qamdo region of Tibet is about 6–7 kg/year, from which it can be inferred that the individual daily consumption of tea is 17 g/day. The European Food Safety Authority (EFSA) sets the minimum human intake of T-2 toxin at 100 ng/kg.b.w [18]. The maximum tolerable daily intake of T-2 toxin is 60 ng/kg.b.w, which was set by the International Health and Food and Agriculture Organization (JEFCA) [22,23]. According to the Chinese adult/child standard body weight and height table, the amount of T-2 toxin ingested from tea in Qamdo, Tibet exceeded the limit set by the European Food Safety Authority and the International Health and Food and Agriculture Organization. Zhao et al. reported that the content of T-2 toxin in grain was not more than 20 μg/kg, which was within the normal range [24]. The content of T-2 toxin in the tea samples we tested was more than 20 times that reported. Moreover, it was 3.96 times more than the allowable FAO standard (100 μg/kg). The T-2 toxin content detected in brick tea in Tibet suggests that there is a great safety risk of mycotoxin intake in brick tea in the Qamdo region of Tibet, which may be one of the causes of the high incidence of Kashin–Beck disease in this region.

## 5. Conclusions

In Tibet, the frequency of brick tea intake in patients with Kashin–Beck disease is higher than that in controls, but there is no significant difference. The high T-2 toxin intake through brick tea may not be the decisive factor inducing Kashin–Beck disease, and there is no significant difference in the diet structure between patients and controls in the affected areas. However, the high T-2 toxin content in brick tea is still the biggest hidden danger to the food safety of people in KBD areas. Therefore, we do not believe that the occurrence of Kashin–Beck disease is the result of a single factor. However, in view of the high levels of T-2 toxin in drinking tea, we have submitted a report to the Chinese government health department, suggesting that the monitoring of T-2 toxin in tea in Tibet should be included in routine monitoring. We hope that the power of public health departments can address the hidden risks of food safety.

## Figures and Tables

**Table 1:** Comparison of drinks and beverages in the Kashin–Beck-disease area.

Drinks and Beverage Category	Patient (*n* = 75)	Controls (*n* = 50)	X^2^	*p*-Value ^1^
Liquor (including all types)	1 (1.33)	0 (0.00)	0.672	0.412
Wine/rice wine/rice wine	1 (1.33)	0 (0.00)	0.672	0.412
Beer	1 (1.33)	0 (0.00)	0.672	0.412
Hulless barley wine	2 (2.67)	0 (0.00)	1.355	0.244
No added water or soda	38 (50.67)	20 (40.00)	1.372	0.241
Artificial sugary drinks include carbonated drinks and commercial teas	35 (46.67)	30 (60.00)	2.137	0.144
Sugar-sweetened beverages, such as soft drinks, fruit drinks, dairy drinks, sweet water and commercial teas, sports or energy drinks	43 (57.33)	37 (74.00)	3.617	0.057
Buttered tea	65 (86.67)	44 (88.00)	0.048	0.827
Tibet sweet tea	40 (53.33)	31 (62.00)	0.918	0.338
Other teas (no added natural green/black/oolong tea, etc.)	38 (50.67)	28 (56.00)	0.342	0.558
Coffee (with or without sugar/milk)	17 (22.67)	21 (42.00) *	5.300	0.021
100% pure juice	17 (22.67)	15 (30.00)	0.847	0.357

^1^ *p*-value represents a significant difference in people who responded between patients and controls. * represents *p* < 0.05, a significant difference in people who responded between patients and controls.

**Table 2:** Contents of T-2 toxin in food samples from KBD areas in Qamdo, Tibet.

Food Type	Survey Sites	*n*	AVE. (μg/kg)
Brick tea	Detong village	5	424 ± 56
	Langcuo village	3	396 ± 24
Zanba powder	Detong village	5	Tr
	Langcuo village	3	Tr
Hulless barley	Detong village	5	Tr
	Langcuo village	3	Tr
Milk residue	Detong village	5	Tr
	Langcuo village	3	Tr
Drinking water	Detong village	6	Tr
	Langcuo village	4	Tr

Tr means the sample result is less than 0.005 ppb, representing no detection or that the detected amount is below the detection line.

## Data Availability

The original contributions presented in the study are included in the article, further inquiries can be directed to the corresponding author.
